# Upregulation of the novel lncRNA U731166 is associated with migration, invasion and vemurafenib resistance in melanoma

**DOI:** 10.1111/jcmm.16987

**Published:** 2022-01-18

**Authors:** Ádamo Davi Diógenes Siena, Isabela Ichihara de Barros, Camila Baldin Storti, Carlos Alberto Oliveira de Biagi Júnior, Larissa Anastacio da Costa Carvalho, Silvya Stuchi Maria‐Engler, Josane de Freitas Sousa, Wilson Araújo Silva

**Affiliations:** ^1^ Department of Genetics Ribeirão Preto Medical School University of São Paulo (USP) Ribeirão Preto Brazil; ^2^ Center for Cell Based Therapy Ribeirão Preto Medical School University of São Paulo (USP) Ribeirão Preto Brazil; ^3^ Department of Clinical and Toxicological Analysis School of Pharmaceutical Sciences University of São Paulo (USP) São Paulo Brazil; ^4^ Institute of Biological Sciences Federal University of Para (UFPA) Belém Brazil; ^5^ Center for Integrative Systems Biology‐CISBi NAP/USP Ribeirão Preto Medical School University of São Paulo (USP) Ribeirão Preto Brazil; ^6^ Institute for Cancer Research Cidade dos Lagos Guarapuava Brazil

**Keywords:** ENSG00000230454, invasion, lncRNAs, melanoma, migration, resistance, U73166, vemurafenib

## Abstract

Our previous work using a melanoma progression model composed of melanocytic cells (melanocytes, primary and metastatic melanoma samples) demonstrated various deregulated genes, including a few known lncRNAs. Further analysis was conducted to discover novel lncRNAs associated with melanoma, and candidates were prioritized for their potential association with invasiveness or other metastasis‐related processes. In this sense, we found the intergenic lncRNA U73166 (ENSG00000230454) and decided to explore its effects in melanoma. For that, we silenced the lncRNA U73166 expression using shRNAs in a melanoma cell line. Next, we experimentally investigated its functions and found that migration and invasion had significantly decreased in knockdown cells, indicating an essential association of lncRNA U73166 for cancer processes. Additionally, using naïve and vemurafenib‐resistant cell lines and data from a patient before and after resistance, we found that vemurafenib‐resistant samples had a higher expression of lncRNA U73166. Also, we retrieved data from the literature that indicates lncRNA U73166 may act as a mediator of RNA processing and cell invasion, probably inducing a more aggressive phenotype. Therefore, our results suggest a relevant role of lncRNA U73166 in metastasis development. We also pointed herein the lncRNA U73166 as a new possible biomarker or target to help overcome clinical vemurafenib resistance.

## 1 INTRODUCTION

Skin cancer is the most common cancer globally, and melanoma is the highly lethal form of this cancer type. Melanoma arises from melanocytes that lost growth and replication control, and melanoma progression phases are well defined according to the stepwise transitions occurring in phenotypic expression from melanocytes to melanoma.^1^, ^2^ The alarming increased incidence of melanoma in most countries during the last decades has been addressed in recent years resulting in a partial decline in the most prevalent regions, such as Australia and Northern America, mainly due to public policy campaigns and new therapies respectively.^3^, ^4^, ^5^ However, in 2021, considering only the United States, there is still a prediction of 101,280 new cases of melanoma in situ, with an estimation of 7180 melanoma‐associated deaths.^6^

Cutaneous melanoma can be classified according to mutations that patients harbour in their cells, and this is known as the Genomic Classification of Cutaneous Melanoma.^7^ This molecular subtype stratification of patients is very relevant because about 50% of melanoma patients have a mutation in the BRAF gene.^8^, ^9^, ^10^ Another reason for BRAF relevance in melanoma is that from all BRAF‐mutants individuals, about 90% harbour the specific V600E mutation^11^, ^12^, ^13^ which activates the MAPK‐ERK pathway constitutively, allowing cells to become self‐sufficient in growth signals and leading to tumour formation.^14^, ^15^

The BRAFV600E relevance in melanoma is also reflected in drugs that have been investigated to target this mutation. The first widely used BRAF‐mutant inhibitor is known as vemurafenib and it initially demonstrated promising results, reducing the risk of death and tumour progression by 63% and 74% respectively.^11^ However, the main obstacles regarding vemurafenib exclusive treatment are the primary resistance that accounts for 20% of the cases and acquired drug resistance.^16^, ^17^ These mechanisms of drug resistance in melanoma are mainly due to the MAPK reactivation with or without the PI3K/AKT pathway activation.^18^ It is well known that mutations in melanoma oncogenes and tumour suppressor genes may result in rupture of diverse pathways involved in cell signalling.^19^ Currently, the clinicians can base their decision in patient's management according to the clinical aspects they present, specific subtype mutations or other relevant biological features to define the melanoma treatment strategies.^20^, ^21^ Considering metastatic melanomas, a combination of strategies can be utilized more effectively than a single therapeutics.^21^

In the last decades, increasing interest in biomarkers and gene therapy using nucleic acids to treat melanoma has revealed its potential application to therapeutics. Small non‐coding RNAs (sncRNAs) were the leading topic of research for many years.^22^, ^23^, ^24^, ^25^ This research field using sncRNAs was improved by developing technologies like microarray profiling, real‐time PCR array and next‐generation sequencing (NGS) technologies.^26^

More recently, long noncoding RNAs (lncRNAs) have begun to be extensively studied and demonstrated a high potential to be used as biomarkers in many cancers and can be helpful for cancer patient management.^27^ The lncRNAs are generally defined as RNA molecules that are 200 nucleotides long and have no protein‐coding ability.^28^ They are expressed in a tissue‐specific manner, participate in a myriad of critical cellular functions and have been implicated as mediators in distinct disease pathogenesis.^29^ In cancer, lncRNAs have initially been pointed out as deregulated transcripts whose expression levels impact normal processes, but recently, they have been associated with functionally relevant alterations in critical cancer processes and pathways.^30^ Therefore, lncRNAs demonstrated an enormous potential to be explored and used as a biomarker in cancer research.^31^, ^32^

An increasing number of novel lncRNAs have been revealed in melanoma, demonstrating their contributions to tumour development. Notably, many of them have great potential to be used as biomarkers and even as therapeutic targets.^33^ They have been implicated in diverse cancer aspects in melanoma as proliferation,^34^ invasion,^35^ metastasis,^36^ migration,^37^ apoptosis^35^ and other tumoural processes. Another reason for the importance of this class of transcripts in melanomagenesis is that they can be more expressed in specific melanoma samples, making them putative biomarker molecules of specific biological aspects regarding melanoma development and staging.^38^

This study explored our previous RNA‐Seq results and found that the novel lncRNA U73166 was deregulated and associated with an invasive profile in melanoma. Moreover, we experimentally verified these findings, and we found that silencing lncRNA U73166 impacts migration, invasion and proliferation in melanoma cells. Our further analysis found an association between the lncRNA U73166 expression and acquired resistance to vemurafenib, suggesting that this lncRNA may play an essential role in melanoma resistance.

## 2 MATERIAL AND METHODS

### 2.1 Melanocytic cell lines

Melanoma cell lines were maintained in Dulbecco's modified Eagle's medium—DMEM (SK‐MEL‐147, SK‐MEL‐5, A375, SK‐MEL‐28) supplemented with 10% (v/v) inactivated foetal bovine serum—FBS (Gibco) or in TU medium (4/5 of MCDB‐153 medium and 1/5 of Leibovitz's L‐15 medium) supplemented with 5 µg/mL of Insulin, 2 mM of CaCl_2_ and 2% FBS (WM164, WM35, WM1552, WM902, WM278, WM793, WM9, WM1617, WM852, 1205lu). Primary melanocytes (MELC 80, MELC 124, MELC 125 and MELC 126) were obtained from patient's foreskins from the University Hospital (Hospital Universitário—HU‐USP). To this end, the project has undergone review and approval by the Ethics Committee of HU (HU no. CEP Case 943/09). These melanocytes were maintained in 254CF medium (Thermo Fisher Scientific), supplemented with HMGS solution (Thermo Fisher Scientific), 200 µM calcium chloride and 2% FBS.

To obtain vemurafenib‐resistant cell lines, we used A375, SK‐MEL‐28 and WM164 cell lines seeded at a low cell density (1×10^4^ cells) in a 60 mm plate, according to Sandri et al., (2016).^39^ Then, these cell lines were treated with increasing doses of vemurafenib in the range 0.5–6 µM every 3 days for a maximum of 6 weeks. It is important to mention that the vemurafenib resistance was previously validated in these cell lines by western blotting and it was confirmed the MAPK pathway reactivation—MEK and ERK phosphorylation levels in vemurafenib presence.^39^, ^40^ These cells were named naïve and resistant cells. The resistant cell lines were continuously refilled with 6 µM vemurafenib every 2–3 days.

All cells were incubated at 37℃ in a humidified atmosphere with 5% CO_2_ and were tested for mycoplasma infection.

### 2.2 Analysis of RNA‐Seq Data and Gene Set Variation Analysis for invasive and proliferative enrichment scores

In our previous work,^41^ we performed Gene Set Variation Analysis (GSVA) with specific gene expression signatures to score our melanoma cell lines and melanoma tissue samples from the TCGA database for invasive and proliferative phenotypes based on their RNA expression profiles.^41^ Here, we evaluated the correlation between the lncRNA U73166 expression level and each of these enrichment scores (proliferative and invasive) in the same set of samples. The expression pattern of lncRNA U73166 in several TCGA tumours and GTEx normal tissues was obtained via gene expression profiling interactive analysis (GEPIA2).^42^ Also, GEPIA2 was utilized to explore gene expression correlations between lncRNA U73166 and RBFOX2, HNRNPA2B1 and SRSF1. To identify RNA‐binding proteins interacting with lncRNA U73166, according to experimental evidence, we utilized CLIP‐Seq data obtained from the starBase v2.0 platform.^43^

### 2.3 RNA extraction and reverse transcription‐quantitative PCR

Total RNA was isolated from cell lines using miRNeasy Mini Kit (QIAGEN) and treated with RQ1 RNase‐Free DNase (Promega) according to the manufacturer's instructions. Then, RNA quantification and control quality were checked in NanoDrop (Thermo Fisher Scientific). Next, we used 500 ng of RNA for cDNA conversion using the High‐Capacity cDNA Reverse Transcription Kit (Thermo Fisher Scientific). For the analysis of lncRNA U73166 gene expression in normal tissues, we used the Human Total RNA Master Panel II (Clontech). The RNA from normal tissues was converted to cDNA, as described for the RNA from cell lines. RT‐qPCR was performed using specific primers for each gene and Power SYBR Green Master Mix (Thermo Fisher Scientific) in a 7500 Fast Real‐Time PCR system (Thermo Fisher Scientific). All procedures were performed following the standard experimental protocol provided by the kit manufacturer.

The designed sequences of primers used for gene expression analysis are as follows: SDHA Forward: 5′‐ CCCGAGGTTTTCACTTCACTG‐3′, Reverse: 5′‐ CCTACCACCACTGCATCAAA‐3′; GAPDH Forward: 5′‐ CTGACTTCAACAGCGACACC −3′, Reverse: 5′‐ TGCTGTAGCCAAATTCGTTG −3′; NEAT1 Forward:5′‐ GTGGAGGAGTCAGGAGGAAT −3′, Reverse: 5′‐ GCTAAGTTCAGTTCCACAAGACC −3′; DANCR Forward: 5′‐ GCTCCAGGAGTTCGTCTCTT −3′, Reverse: 5′‐ CAACAGGACATTCCAGCTTC −3′; U73166 Forward: 5′‐ GCGGTCCTCATCTCTACCAT‐3′, Reverse: 5′‐ GTAATTCCAGACCCCTGTGG‐3′; TBP Forward: 5′‐ AGCTGTGATGTGAAGTTTCC‐3′, Reverse:5′‐ TCTGGGTTTGATCATTCTGTAG‐3′.

### 2.4 Subcellular fractionation

To obtain RNA from A375 cell line subcellular fractions, we utilized the Ambion PARIS kit (Thermo Fisher Scientific) following instructions provided by the manufacturer. Briefly, melanoma cells were trypsinized, and a total of 1x10^6^ cells was pelleted in a microfuge tube. These cells were submitted to cell fractionation and centrifugation at 4°C, resulting in partitioned nuclear and cytoplasmic fractions. The supernatant was relocated to another microfuge tube, and the pellet remained in the same tube. The following steps included each subcellular lysate being submitted to column binding and washing, and each fraction of nuclear RNA and cytoplasmic RNA was collected in different tubes. The subsequent steps included DNase treatment, RNA quality control, RNA quantification and cDNA production, as mentioned above.

### 2.5 Generation of shRNA constructs and lentiviral transduction

First, we used the lncRNA U73166 FASTA sequence with the best ENSEMBL support to design three shRNAs using the web‐based tool for siRNA selection from Whitehead Institute (http://sirna.wi.mit.edu/). Then, the commercially obtained sense and antisense oligos (Exxtend Oligos) were annealed in a thermocycler under the following conditions: 95°C for 4 min, 70ºC for 10 min, and then let for a slow cooling down for 12 h. These double‐stranded DNA oligos were purified with the Wizard DNA Purification Kit (Promega). We used 6 µg of the lentiviral vector pLKO.1 (Addgene) for digestion with *Age*I and *Eco*RI restriction enzymes (New England Biolabs) according to the manufacturer's instructions. Digestion was confirmed by electrophoresis in an agarose gel. Subsequently, the digested plasmid‐corresponding band was gel purified using the Wizard DNA Purification Kit (Promega) and then used for a ligation reaction, which was carried out with 20 ng of digested pLKO.1 and double‐stranded oligo DNAs using T4 DNA ligase (New England Biolabs) and proper buffer at 16°C overnight. The resulting ligation products were used to transform DH5α competent cells (Thermo Fisher Scientific) through thermal bacterial transformation. Positive colonies were cultivated at 37°C overnight in LB broth, and the bacteria were pelleted for plasmid DNA extraction. These plasmids containing shRNAs were submitted to Sanger sequencing in the ABI 3500xL Genetic Analyzer equipment (Thermo Fisher Scientific) and the correct insertion of shRNAs into pLKO.1 vector and their sequences were confirmed. Confirmed clones were expanded and, pLKO.1‐shRNAs constructs were purified using the Qiagen Plasmid Midiprep kit (Qiagen).

For lentiviral production, we used HEK293T cell lines for transfection in a 6‐well plate. We used 250ng per well of lentiviral envelope pMD2.G (Addgene) plasmid, 125 µg of psPAX2 (Addgene) packaging plasmid, 1.25 µg of each pLKO.1‐shRNA construct and PEI MAX (Polysciences, Inc) at a concentration of 1 mg/ml. Later, lentiviral particles were collected from HEK293T culture media and utilized to transduce A375 melanoma cells. Transfected cells expressing shRNAs against lncRNA U73166 were selected with 1 μg/mL puromycin for at least 3 days. To certify that only cell lines expressing PLKO‐shRNAs survived, non‐transduced A375 was used as a control and treated with identical puromycin concentrations during the same period. The cells transduced with the two selected shRNAs were named shU73166#1 and shU73166#3. The sequences of the oligonucleotides used for these constructs are the following: shU73166#1 Forward: 5’ – CCGGCCTGTCAATTCAGCCTTGTCTCGAGACAAGGCTGAATTGACAGGTTTTTG – 3’, shU73166#1 Reverse: 5’ – AATTCAAAAACCTGTCAATTCAGCCTTGTCTCGAGACAAGGCTGAATTGACAGG – 3’, shU73166#3 Forward: CCGGCATTCATCAACCCTCAGGACTCGAGTCCTGAGGGTTGATGAATGTTTTTG – 3’, shU73166#3 Reverse: AATTCAAAAACATTCATCAACCCTCAGGACTCGAGTCCTGAGGGTTGATGAATG – 3’. Cells transduced with the empty pLKO.1 vector were used as negative control (shPLKO#NC).

### 2.6 Proliferation assay

A375 cells were seeded at a density of 5.000 cells/well in 96‐well plates and cultured in DMEM medium. The first measurement (time =0h) was performed 3h after platting to allow cells to attach to the bottom of the wells. The following measurements were performed according to the continuous experiment time (24h, 48h, 72h and 96h). At each specified time point, the DMEM medium was removed from plates, and cells were fixed with 70% ethanol for 10 min at room temperature. Then, ethanol was removed, crystal violet (0.5%) was added, and the plates were incubated at room temperature for 15 min. Subsequently, fixed cells were washed six times with water, and the plates were used after they had completely dried. After the addition of 100 µl of 10% acetic acid per well, the plates were incubated for 30 min at room temperature. Finally, absorbance values at 540 nm were measured in the FLUOstar Omega plate reader (BMG Labtech). All the experiments were performed in five replicates.

### 2.7 Transwell migration and invasion assays

In vitro cell migration was performed using Thincert Cell Culture Insert For 24 Well Plates (Greiner Bio‐One). Briefly, a volume of 600 µl of cell suspension (1 × 10^4^ cells) in DMEM serum‐free medium was added into the upper chambers. A volume of 600 µL DMEM medium with 10% FBS without antibiotic was added in the lower chamber to induce cell migration. After 24 h incubation, the medium was removed, and migrated cells were fixed with 4% paraformaldehyde and stained using 0,5% crystal violet solution. The non‐invading cells were removed from the insert's upper surface using a cotton swab, and five random fields were photographed using the inverted microscope IX71 system (Olympus). Images were later processed, quantified and analysed using ImageJ software.

In vitro cell invasion was conducted using BioCoat Matrigel Invasion Chamber assay (Corning). The invasion chamber was removed from the freezer and rehydrated with DMEM medium at 37°C. DMEM was added to the insert's interior and the bottom of wells 2 h before plating the cells. The following steps were performed as described above for the migration assay. All the experiments were performed in triplicate.

### 2.8 Wound healing assay

A375 melanoma cells were plated in 6‐well plates at a density of 5x10^4^ cells per well and cultivated for 6 h until they reached 90% of confluence. After that, the cell monolayer was gently scratched using a sterile 200 µl pipette tip in a continuous movement. Each well was washed twice with 37°C pre‐warmed 1X PBS, and 5 ml of DMEM medium were added to each well. All the plates remained in controlled conditions of 37°C and 5% CO^2^. The images of specific points in each well were taken at 0h, 24 and 48 h. We used ImageJ software (Bethesda, MD, USA) to process images acquired and for quantitative analysis. The quantification of the relative wound area closure is presented in relative units. Results represent the mean of three measurements of each wounded area obtained in three independent experiments.

### 2.9 Statistical and image analysis

Statistical analyses were carried out using the R platform and the PRISM software package (version V.6.01, GraphPad Software). Values of *p* ≤ 0.05 were considered statistically significant. ImageJ software was used to process and perform measurements in wound healing experiments, invasion and transwell migration assays.

## 3 RESULTS

### 3.1 The novel lncRNA U73166 is upregulated in melanoma and its expression is correlated with an invasiveness signature

**FIGURE 1:**
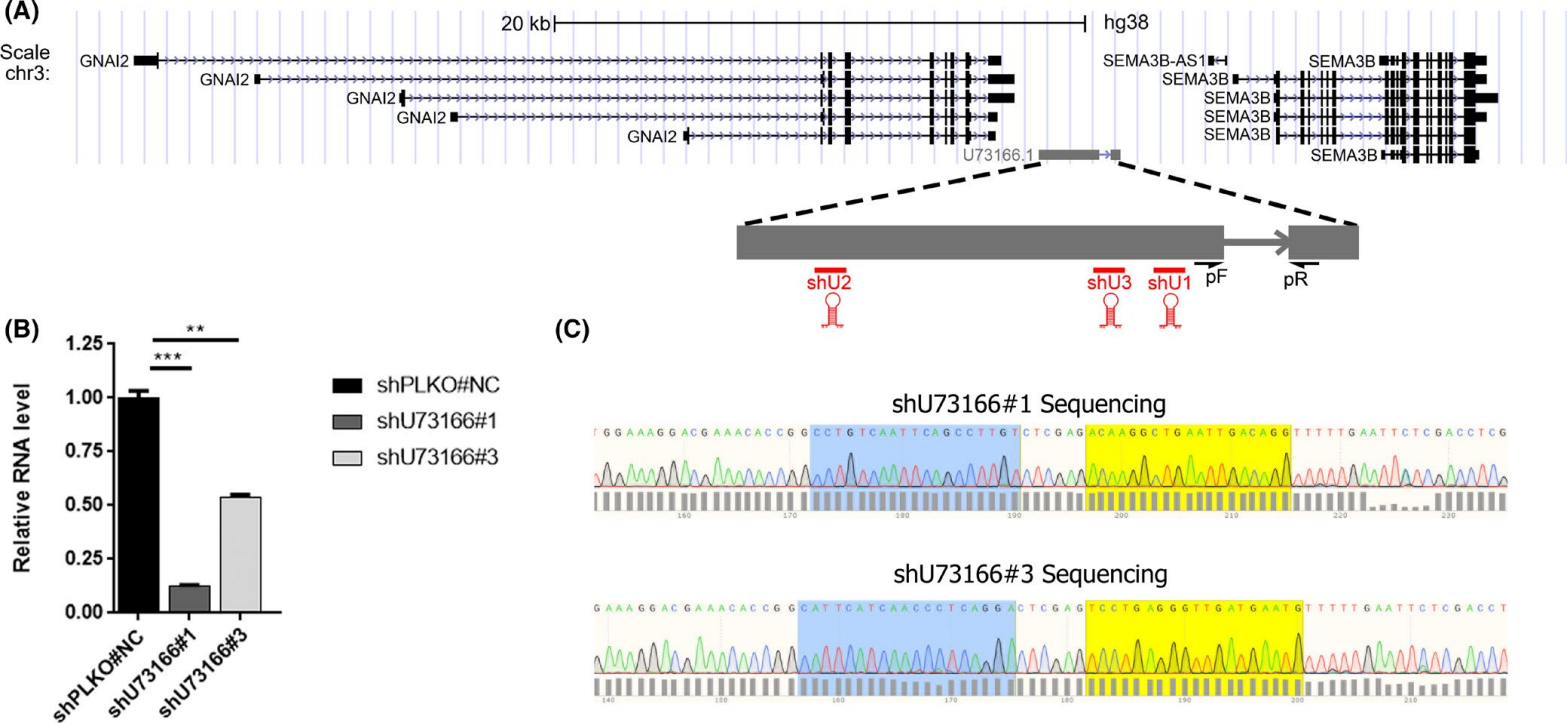
Genomic context of the lncRNA U73166 and experimental approach utilized. (A) UCSC genome browser image depicting localization of lncRNA U73166 and neighbouring coding genes. The transcript is zoomed (dashed lines) to demonstrate that lncRNA U73166 is composed of two exons. Red lines illustrate the regions targeted by shRNAs (shU1 = shU73166#1, shU2 = shU73166#2, and shU3 = shU73166#3). Black arrows indicate the designed primers for RT‐qPCR experiments (pF =primer forward and pR =primer reverse). (B) Silencing efficiency experiment showing that designed shRNAs can effectively reduce expression levels of the lncRNA U73166. Expression levels were determined by RT‐qPCR according to the 2^−ΔΔCt^ method and using the expression of TBP for normalization, as loading control. The Student's *t*‐test was performed to compare differences between experimental groups. Data are presented as mean ± SD from three independent experiments performed in triplicate. (C) Sanger sequencing from shU73166#1 and shU73166#3 demonstrating correct orientation and insertion of the shRNAs into the plasmid PLKO.1. Blue and yellow highlighted areas represent sense and antisense sequences from each shRNA respectively. *p  ≤ 0.05, **p  ≤ 0.01, ***p  ≤  0.001

**FIGURE 2:**
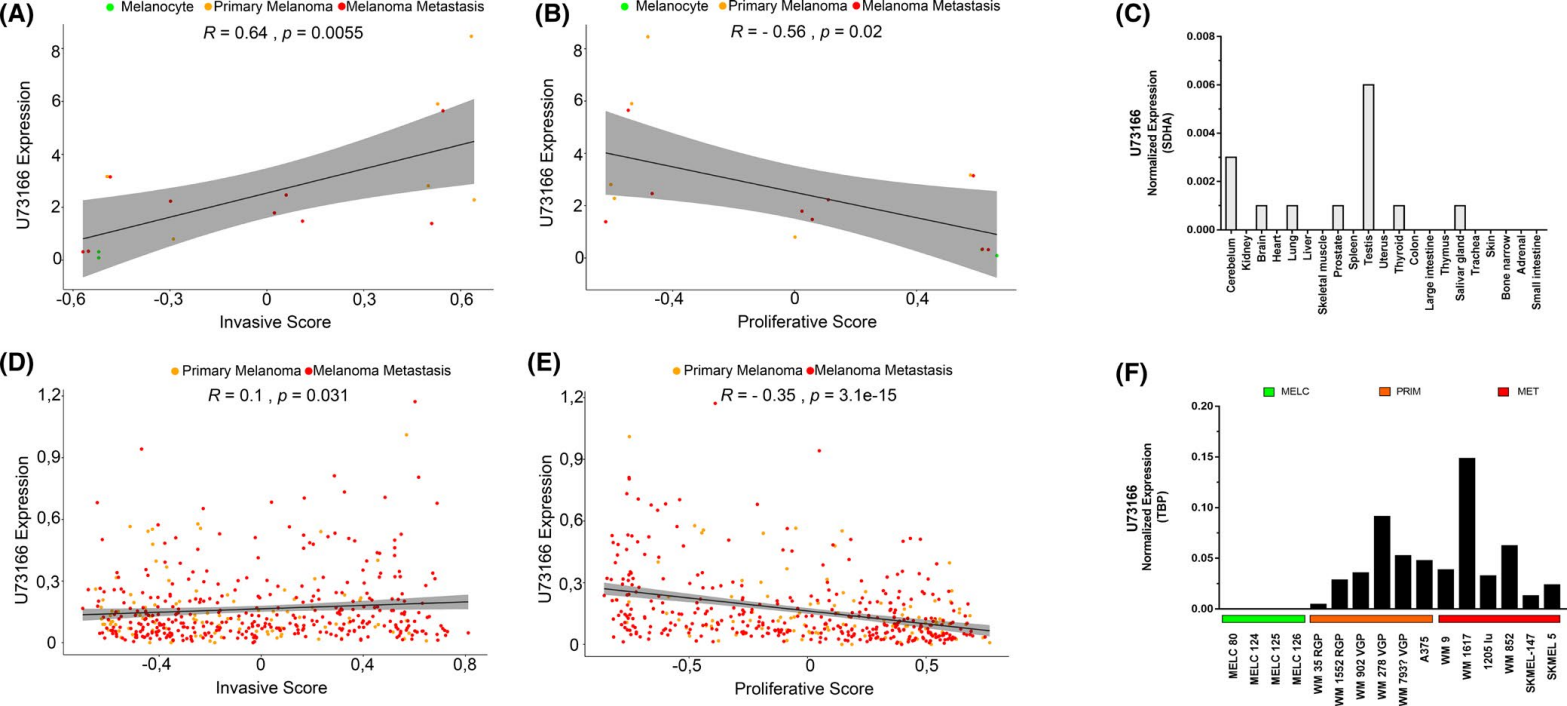
Gene set variation analysis of invasive/proliferative profile and lncRNA U73166 gene expression in normal tissues and melanocytic cells. (A) Positive correlation between lncRNA U73166 gene expression and invasive score in melanocytic cell lines. (B) Negative correlation between lncRNA U73166 gene expression and proliferative score in melanocytic cell lines. (C) Gene expression of the lncRNA U73166 in a panel of normal tissues showing higher expression in testis. SDHA was used as internal control and the 2^−∆Ct^ method was utilized for normalization and analysis. (D) Positive correlation between lncRNA U73166 gene expression and invasive score in TCGA melanoma samples. (E) Negative correlation between lncRNA U73166 gene expression and proliferative score in TCGA melanoma samples. (F) Gene expression of the lncRNA U73166 in melanocytes, primary and metastatic melanoma cell lines. TBP was used as internal control and the 2^−∆Ct^ method was utilized for normalization and analysis

Our previous work using a melanoma progression model composed of melanocytes, primary and metastatic melanoma samples indicated several s deregulated genes, including a few known lncRNAs.^41^ Further analysis was carried out to discover novel lncRNAs impacting melanoma development, and candidates were prioritized due to their potential association with invasiveness. In this sense, we identified the intergenic lncRNA U73166 (ENSG00000230454) located in the region between the protein‐coding genes SEMA3B and GNAI2 (Figure 1A), and this transcript was selected to be further investigated. We observed that lncRNA U73166 was upregulated in metastatic melanoma cell lines compared to normal melanocytes (log fold change: 4.32; Adjusted *p*‐value: 0.038). This lncRNA demonstrated a significant positive correlation with invasiveness score in melanocytic cell lines and melanoma samples from TCGA consortium (*r* = 0.64, *p* = 0.0055 and *r* = 0.1, *p* = 0.031 respectively) (Figure 2AandD). In the same analysis, it was possible to verify that lncRNA U73166 demonstrated a significant negative correlation with proliferation score in melanocytic cell lines and TCGA tumours (r = −0.56, *p*‐value =0.02 and *r* = −0.35, *p*‐value =3.1e‐15 respectively) (Figure 2BandE). According to the ENSEMBL database, the most reliable version of U73166 lncRNA is a 2625 nucleotides long sequence, encompassing two exons, and up to now, there is no more information in the scientific literature regarding this transcript.

### 3.2 The lncRNA U73166 is expressed in a melanoma‐testis pattern

We searched the expression profile of the lncRNA U73166 in a public data platform,^42^ and noted that its expression is higher in testis than in any other normal or tumoural tissues (Figure S1A and 1B). We decided to experimentally validate these results assessing lncRNA U73166 expression in several melanocytic cell lines and in a panel of human RNA samples from normal tissues. We confirmed that lncRNA U73166 is more expressed in normal testis in comparison to other normal tissues (Figure 2C) and, that it is highly expressed in melanoma cells compared to melanocytes, although not presenting a specific trend of enrichment to a particular melanoma progression phase (Figure 2F). In addition, according to the data available in TCGA repository, the expression level of lncRNA U73166 is not significantly different among molecular subgroups of melanoma samples (Figure S2A) nor between primary and metastatic melanomas (Figure S2B).

### 3.3 Downregulation of lncRNA U73166 impacts proliferation and invasion in melanoma cells

**FIGURE 3:**
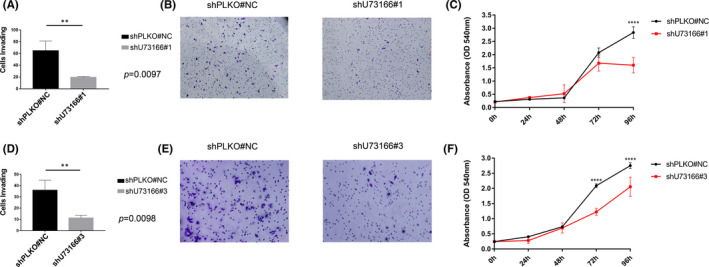
Effect of lncRNA U73166 knockdown on cell invasion and proliferation. (A and D) Control samples demonstrated a significantly higher number of cells invading than silenced cells. Student's t‐*t*est was performed to compare differences between experimental groups. Data are presented as mean ± SD from three independent experiments performed in triplicate. (B and E) Representative images depicting the differences between control cells (higher invasive capacity) and cells with lncRNA U73166 silenced (reduced invasion). (C and F) Quantitation of the proliferation assay showing that after 48 h the control samples presented higher rates of proliferation than U73166‐silenced cells. (A, B and C) images are from shU73166#1‐induced silencing, and (D, E and F) images are from shRNAU73166#3‐induced silencing. Statistical analysis was based on the ANOVA test. Data are presented as mean ± SD. *p ≤ 0.05, **p ≤ 0.01, ***p ≤ 0.001

To experimentally validate our previous GSVA results indicating a positive and a negative correlation of the lncRNA U73166 expression with invasiveness and with proliferation, respectively, we evaluated the effects of lncRNA U73166 knockdown, using short hairpin RNA (shRNA), in melanoma cell's abilities for migration, invasion and proliferation in melanoma cell line A375. For that, three shRNAs were designed and, two of them (shU31766#1 and shU73166#3) were used to target lncRNA U73166 in the melanoma cell line A375 (Figure 1A). The shU31766#1 and shU73166#3 were selected according to silencing efficiency—68% and 51% respectively (Figure 1B)—and their successful cloning and sequence confirmation (Figure 1C). In agreement with our GSVA result, after U73166 knockdown, we observed a significantly reduced invasion ability of A375 melanoma cells (Figure 3A,B,DandE). However, regarding proliferation, we observed the opposite. As our GSVA result indicated a negative correlation between U73166 expression level and the proliferative score, we had expected to see an increase in proliferation upon U73166 knockdown, but instead, we observed significantly higher proliferation rates in controls than in silenced cells (Figure 3CandF), which may imply lncRNA U73166 interfering positively in this process. Therefore, as our primary interest is related to metastasis, we decided to check if lncRNA U73166 downregulation could have an impact on other responses that are more reflective of migratory behaviour.

### 3.4 lncRNA U73166 silencing impacts cell migration

**FIGURE 4:**
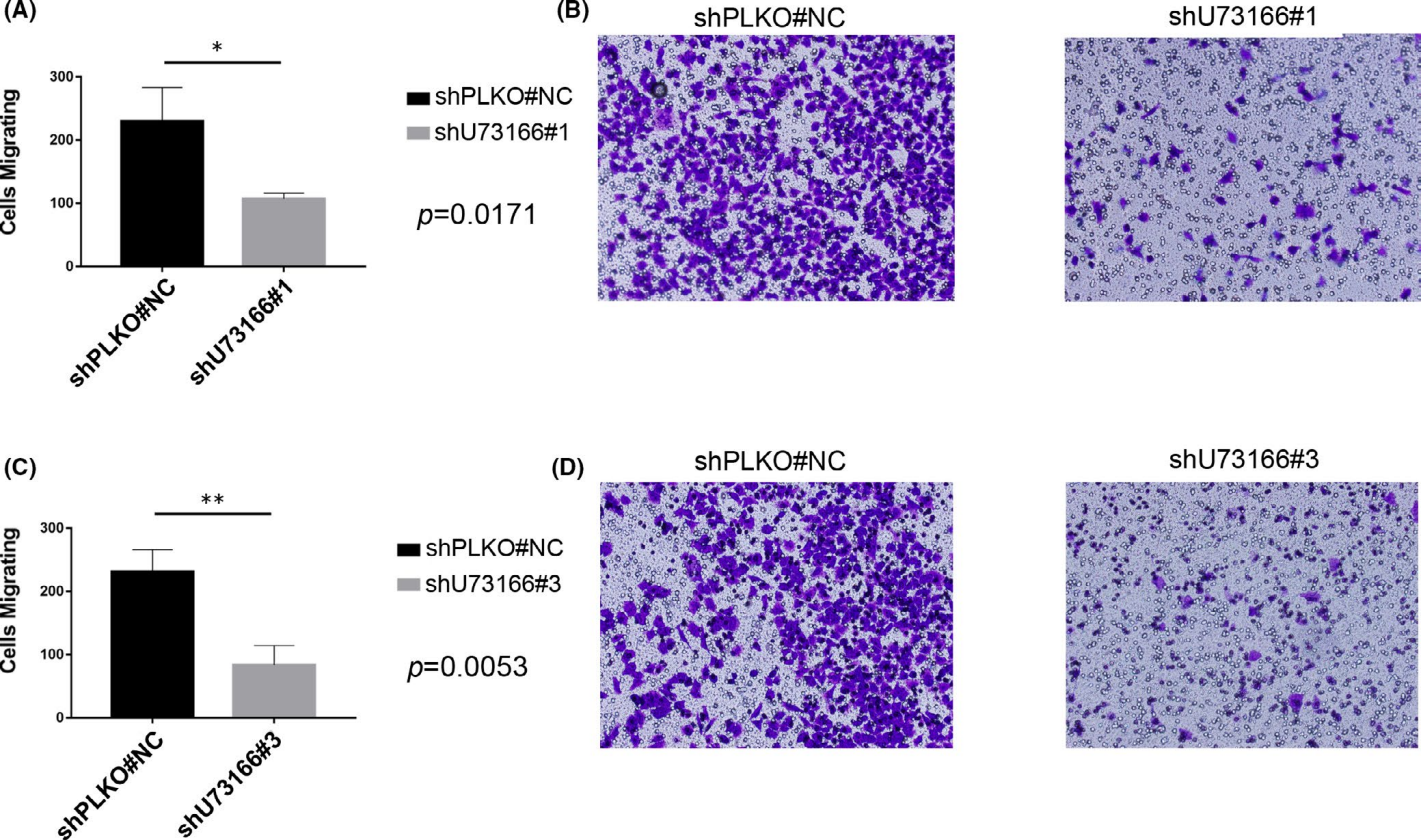
Effect of lncRNA U73166 knockdown on cell migration. (A and C) Quantitation of migration assays showing significantly reduced number of migrating cells after lncRNA U73166 knockdown. (B and D) Representative images illustrating the higher migration rates in control cells than in lncRNA U73166 silenced cells. (A and B) Representative images from shU73166#1‐mediated silencing, and (C and D) representative images from shU73166#3‐mediated silencing. *p ≤ 0.05, **p ≤ 0.01, ***p ≤ 0.001.

**FIGURE 5:**
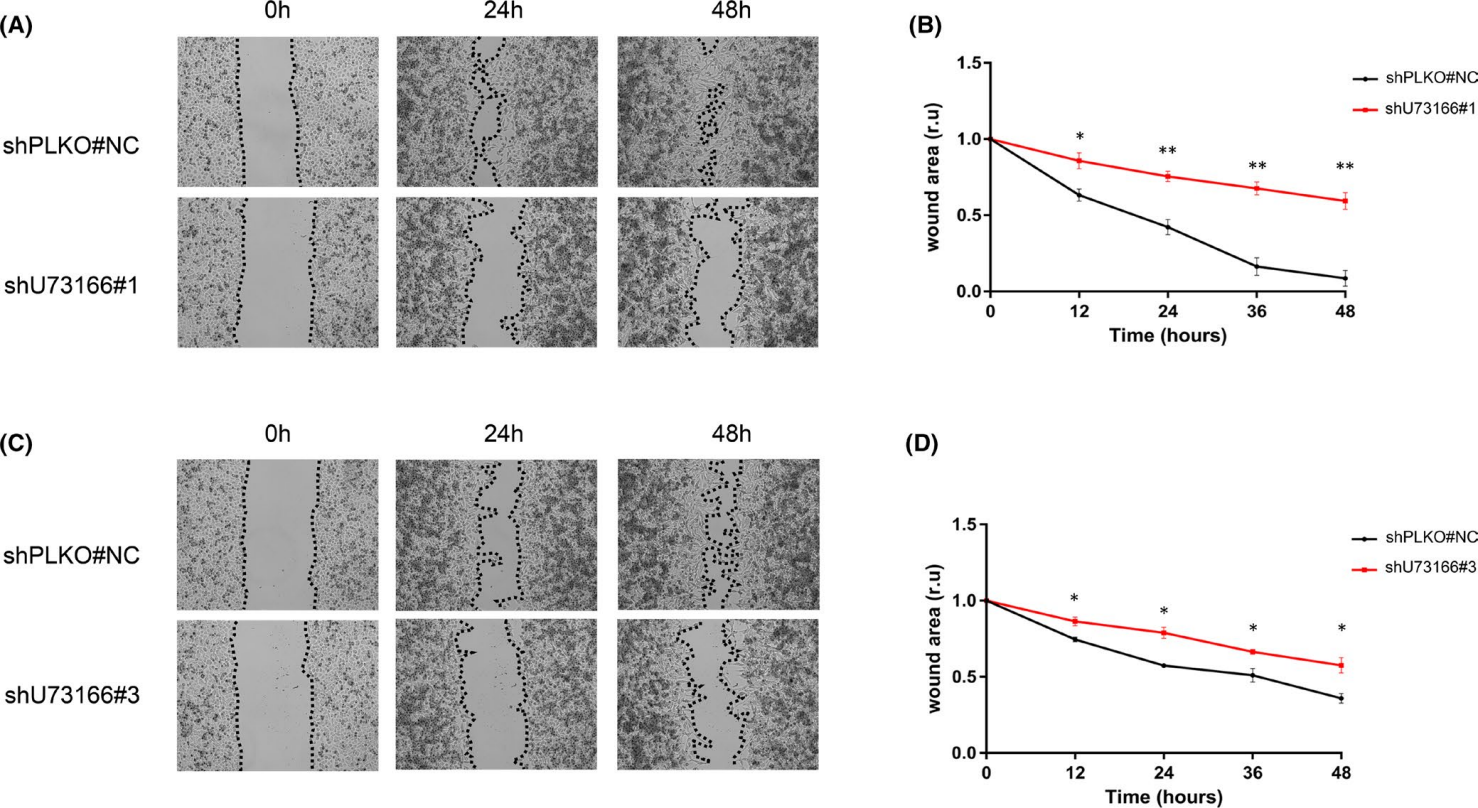
Effect of lncRNA U73166 knockdown on collective cell migration assessed by wound healing assay. (A and C) Representative images showing more pronounced decrease in wound area over time in control samples when compared with silenced cells. (B and D) Graphs with the wound healing assay measurements showing that significantly higher relative areas were covered by control cells than by silenced cells. Student's *t*‐test was performed to compare differences between experimental groups. Data are presented as mean ± SD from three independent experiments performed in triplicate. *p ≤ 0.05, **p ≤ 0.01, ***p ≤ 0.001

Further analysis was conducted using different assays to evaluate if lncRNA U73166 was associated with migration. Using transwell migration assay, we found that the number of migrating control cells was significantly higher than for silenced cells (Figure 4). This result indicates that lncRNA U73166 may induce the migratory phenotype of melanoma cells. So, we decided to test if lncRNA U73166 reduced expression levels could also influence collective cell migration, and for that, we performed the wound healing (‘scratch’) assay. Interestingly, we found that control cells presented higher migration rates than silenced cells (Figure 5). This result indicates that collective melanoma cell migration could also be affected by reduced lncRNA U73166 gene expression. Therefore, we could infer that lncRNA U73166 gene expression can impact melanoma cells in their cell migration abilities.

### 3.5 Subcellular fraction analysis demonstrates nuclear enrichment of the lncRNA U73166

**FIGURE 6:**
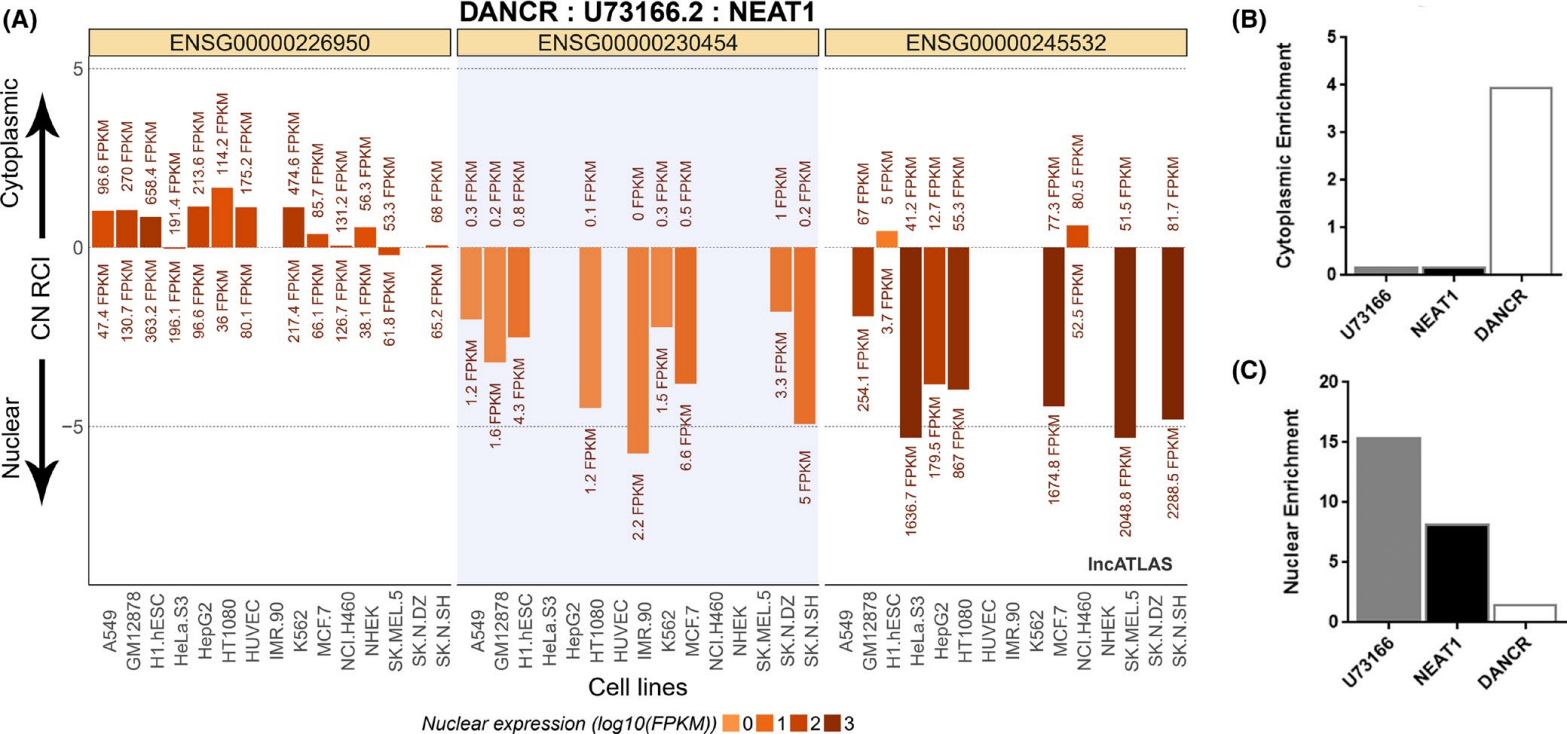
Subcellular localization and enrichment of lncRNA U73166. (A) Plot obtained from lncATLAS showing the subcellular localization of lncRNA U73166 for diverse cell lines (highlighted area in the centre), except to the unique melanoma cell line present in the dataset (SK‐MEL‐5). Markers for subcellular localization comparison are showed on the left side, cytoplasmic lncRNA marker DANCR, and on the right side the nuclear‐retained lncRNA NEAT1. (B) Cytoplasmic fraction of A375 cell line showing enrichment of cytoplasmic marker DANCR, and scarce enrichment of nuclear marker NEAT1 and lncRNA U73166. (C) Nuclear fraction of A375 cell line showing low enrichment of cytoplasmic marker DANCR and high enrichment levels of nuclear marker NEAT1 and lncRNA U73166. The 2^−∆Ct^ method was utilized for normalization and analysis

It is well known that many lncRNAs accumulate into specific subcellular compartments.^44^ Thus, we decided to check if the lncRNA U73166 is enriched in cytoplasmic or nuclear compartments. This analysis was first performed using public data derived from ENCODE and available in lncATLAS.^45^ The majority of available cell lines data demonstrated a clear lncRNA U73166 expression pattern indicative of nuclear enrichment in several cell lines (Figure 6A). However, the lncRNA U73166 data were not available for the unique cell line representative from melanoma (SKMEL5) in this dataset. (Figure 6A). Due to this intriguing result, we decided to check if we could detect subcellular‐enriched levels of U73166 in one of our melanoma cell lines. For that, we separated cytoplasmic and nuclear fractions from melanoma cell line A375 and measured by RT‐qPCR the levels of the lncRNA U73166 in each of these compartments. We found a high enrichment of the lncRNA U73166 in the nuclear fraction compared to the cytoplasmic fraction (Figure 6BandC). Surprisingly, our results demonstrated a higher nuclear enrichment for U73166 than the observed for the nuclear‐enriched marker lncRNA NEAT1, commonly used in this type of analysis as a positive control for nuclear enrichment. (Figure 6C). It is important to mention that we utilized the same lncRNA gene markers for subcellular compartment enrichment as the public database.

### 3.6 The lncRNA U73166 expression level is associated with vemurafenib resistance in BRAFV600E mutants

**FIGURE 7:**
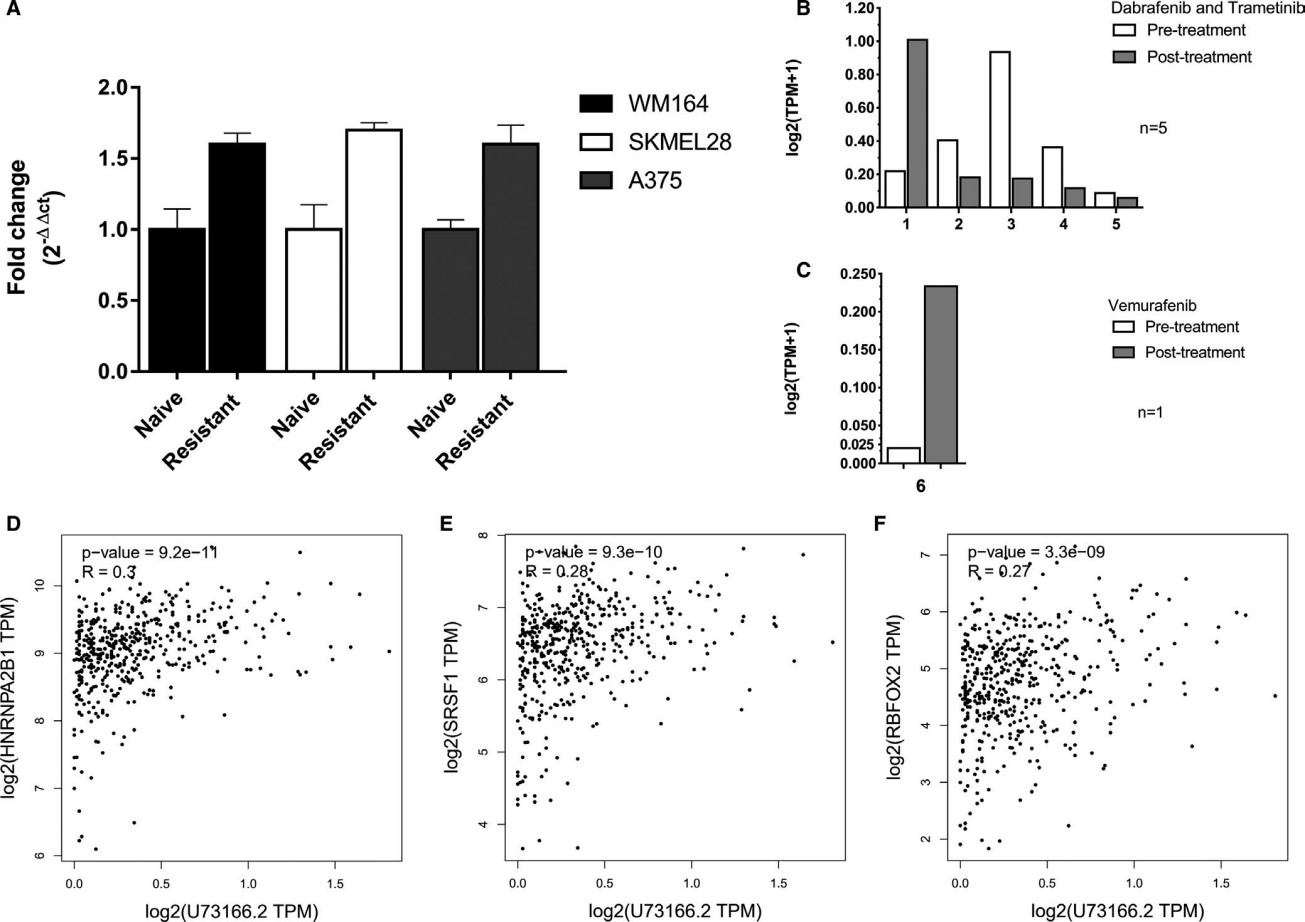
Vemurafenib resistance in melanoma cell lines, patient samples and lncRNA U73166 correlation of gene expression with experimentally verified RBP. (A) RT‐qPCR from three cell lines pairs (naïve and resistant) showing higher fold change of lncRNA U73166 in resistant cell lines compared with naïve cells. The data are presented as means ± SD from three independent experiments performed in triplicate. (B) Five melanoma patients treated with a combination of Dabrafenib +Trametinib demonstrated unequal levels of lncRNA U73166 gene expression. (C) A patient that was treated only with vemurafenib showed 10 times elevated levels of lncRNA U73166 after acquiring drug resistance. (D) Positive and significant correlation of gene expression between HNRNPA2B1 and lncRNA U73166. (E) Positive and significant correlation of gene expression between SRSF1 and lncRNA U73166. (F) Positive and significant correlation of gene expression between RBFOX2 and lncRNA U73166

To check if the lncRNA U73166 could be associated with melanoma drug resistance, we utilized three melanoma cell lines that harbour the BRAFV600E mutation. These original cell lines (naïve) were treated with increasing concentrations of vemurafenib to induce them towards acquired drug resistance, and then they were labelled as resistant. Our results demonstrated that the resistant cell lines express significantly higher levels of the lncRNA U73166 than the naïve cells (Figure 7A). This result indicates that vemurafenib resistance may be associated with increased lncRNA U73166 transcript levels. Moreover, we used public RNA expression data^46^, ^47^ from a patient treated with vemurafenib who underwent melanoma biopsy before and after acquiring vemurafenib resistance. We compared the data from this patient with five other patients treated with different anti‐melanoma drugs. The results showed that in all the other five patients submitted to treatments not including vemurafenib the lncRNA U73166 expression levels did not correlate with resistance (Figure 7B). However, for the patient who developed resistance after vemurafenib treatment lncRNA U73166 gene expression was 10 times higher in melanoma cells after than before treatment. (Figure 7C). These results could implicate a possible correlation between melanoma resistance and higher levels of lncRNA U73166.

### 3.7 lncRNA U73166 interacts with a diverse group of proteins and is a potential mediator of deregulated RNA processing in cancer

To gain additional insight regarding how lncRNA U73166 modifies BRAFV600 expression, we probed for protein with which U73166 may interact at the transcriptional level in the nucleus using the CLIP‐Seq public database.^43^ We found many RNA‐binding proteins (RBP) that directly interact with lncRNA U73166 (Table S1). We ranked all these RBP interacting partners, and the top 10 well‐supported genes (with at least 4 CLIP‐Seq supporting experiments) were shortlisted. From that list, we analysed the correlation between their mRNA levels and the lncRNA U73166 expression. Notably, HNRNPA2B1 (*R* = 0.3 and *p*‐value =9.2e−11), SRSF1 (*R* = 0.28 and *p*‐value = 9.3e−10), and RBFOX2 (*R* = 0.27 and *p*‐value = 3.3e−09) demonstrated higher coefficient correlation and significance with lncRNA U73166 gene expression (Figure 7D,EandF respectively).

## 4 DISCUSSION

Recently, many potential regulatory lncRNAs have been identified, and this field shows many transcripts with a wide range of functions. In cancer‐related studies, lncRNAs have several roles associated with many aspects of carcinogenesis.^30^ Due to the specific expression pattern that lncRNAs demonstrate, their importance is being mentioned in an increasing number of studies in the last decades. Indeed, some of them have already been described as specific biomarkers,^48^ and other cancer‐specific lncRNAs probably will be revealed in the following years. The studies in this area will enable researchers to tackle many challenges in cancer research that are difficult to address with current knowledge and approaches.

Our findings revealed, for the first time, that the lncRNA U73166 is expressed in a melanoma‐testis pattern, which resembles the antigen‐testis pattern, particularly interesting in the search for molecules as potential biomarkers and targetable molecules in the tumour.^49^, ^50^ Thus, the novel lncRNA U73166 can be a valuable transcript to be explored in melanoma assessment and treatment in the future.

Using knockdown experiments, we could test if our previous bioinformatics findings associating U73166 levels with an invasive expression profile would be experimentally validated. Our results demonstrated that cells presented reduced invasive potential after shRNA‐mediated silencing of the lncRNA U73166. It is worthy of mentioning that the melanoma invasive state is mediated by key regulators, as AP1 and TEADs, and alterations in the expression of genes involved in invasion are also associated with an increase in patient's therapy resistance, including BRAF inhibitors.^51^ Moreover, using an insert‐based migration experiment, we found a higher level of cells migrating in control samples than cells in which lncRNA U73166 had been silenced. We also found that in wound healing assay, silenced cells presented decreased ability to cover the scratch area, demonstrating that lncRNA U73166 also affects collective cell migration. As the expression of lncRNA U73166 was positively associated with an invasiveness expression profile (bioinformatics supported), and with migration, and invasion (experimentally supported), our findings suggest a role of lncRNA U73166 in melanoma invasive and migratory phenotypes.

Surprisingly, the proliferation rate—that was negatively correlated with lncRNA U73166 gene expressions in bioinformatics analysis—was found elevated in control cells compared to silenced cells in our experiments. The disparities between the results of proliferation experiments versus bioinformatics analysis may reflect idiosyncrasies from the different cell lines and TCGA samples used. We believe that the A375 melanoma cell line used in this study does not comprise all the different aspects that other melanoma cell lines may harbour, such as specific mutations (other than BRAFV600E) or interactions within the different sites to where t they metastasize to (for metastatic cell lines). Furthermore, it is possible to observe that proliferation showed significant differences between control and silenced cells only after 48h (Figure 3C,F), whereas the wound healing experiment was conducted only up to 48h. Thus, it is possible that when synchronized, silenced cells may show a robust invasive phenotype in the first hours and then switch to a more proliferative profile. That is an important point due to the fine‐tuning way that ncRNAs may regulate cell behaviour and demonstrates that they can present different roles in a timescale manner.

It is well known that the BRAFV600E mutation is present in approximately 50% of all the melanoma cases. This alteration constitutively activates the MAPK/ERK pathway and is involved in increased proliferation. Although there are available drugs targeting this alteration (vemurafenib), treatment shortly fails because cells acquire resistance, mainly shifting to CRAF and ARAF activation. Thus, understanding how resistance acts is essential, and recent studies on lncRNA could shed light on this process. Therefore, using different melanoma cell lines that harbour BRAV600E mutation, we could show that lncRNA U73166 seems to contribute to drug resistance as it showed higher levels in vemurafenib‐resistant cell lines when compared with paired naïve cells. Similarly, samples originating from patient biopsies before and after resistance demonstrated higher levels of lncRNA expression in melanoma when the patient acquired vemurafenib resistance. These results could provide essential information that lncRNA U73166 can impact and be a potential biomarker of resistance, and its levels could be essential in stabilizing acquired drug resistance.

Subcellular fraction analysis provides important insight regarding how changes in lncRNA expression levels modulate cell behaviour. Our findings revealed enrichment of the lncRNA U73166 in the nuclear fraction, indicating that its function could be related to gene expression regulation at the transcriptional level or other nuclear relevant events. Analysis in a public database of interactions supported by CLIP‐seq data allowed us to retrieve proteins that directly interact with lncRNA U73166 and many of them participate in RNA processing and splicing events. Some of these proteins have recently been revealed as having key roles in migration, invasion, epithelial‐mesenchymal transition (EMT), metastasis, and have been associated with other lncRNAs in other types of cancer.^52^, ^53^, ^54^, ^55^, ^56^ It is well known that in the nuclear compartment, lncRNAs may act as a scaffold and be associated with proteins working in the gene regulation process. According to our analysis potential protein partners interacting with lncRNA U73166 included HNRNPA2B1, SRSF1 and RBFOX2, which demonstrated a robust positive expression level correlation with lncRNA U73166. While insufficient information regarding melanoma was found in the literature, we found that RBFOX2 and HNRNPA2B1 have been established to regulate tumour development—primarily through EMT‐related processes—and in pancreatic cancer, HNRNPA2B1 acts through the ERK/Snail pathway.^54^, ^57^, ^58^ It is essential to mention that ERK1/2 are critical regulators of the MAPK signalling pathway and act as both downstream targets and upstream regulators (negative feedback) of the A/B/C‐RAF kinases.^59^ Therefore, a direct interaction between lncRNA U73166 and RBPs such as HNRNPA2B1 and their correlated expression may indicate a role for lncRNA U73166 in the regulation of the MAPK signalling pathway. However, confirmation of this hypothesis will require further investigation. If confirmed, the relationship between lncRNA U73166 and BRAFV600E mutants may represent an important finding as this transcript may be an essential mediator in this cancer signalling pathway. Additionally, to our results, further analysis in the future should include the silencing of lncRNA U73166 using ASO (antisense oligos) to assess if the phenotypic changes remain or are more pronounced.

Our findings showing that silencing of lncRNA U73166 impacts melanoma tumoural processes and that vemurafenib‐resistant cells have significantly higher levels of lncRNA U73166 could be helpful for patient assessment and therapeutic management. In the future, these results may be beneficial to melanoma research and therapeutics, contributing, for example, to expand the field of lncRNAs biomarkers for this tumour or to indicate better approaches for drug‐resistance monitoring and potential molecular targets for improvement of melanoma therapy.

## CONFLICT OF INTEREST

All the authors declare no competing interests.

## AUTHOR CONTRIBUTIONS

**Ádamo Davi Diogenes Siena:** Conceptualization (lead); Methodology (lead); Validation (lead); Writing‐review & editing (lead). **Isabela Ichihara de Barros:** Methodology (equal); Writing‐review & editing (equal). **Camila Baldin Storti:** Methodology (equal); Writing‐review & editing (equal). **Carlos Alberto Oliveira de Biagi Júnior:** Methodology (equal); Writing‐review & editing (equal). **Larissa Anastacio da Costa Carvalho:** Conceptualization (equal); Methodology (equal); Resources (equal); Writing‐review & editing (equal). **Silvya Stuchi Maria‐Engler:** Conceptualization (equal); Supervision (equal); Writing‐review & editing (equal). **Josane de Freitas Sousa:** Supervision (equal); Writing‐review & editing (equal). **Wilson Araújo Silva, Jr:** Conceptualization (equal); Resources (lead); Supervision (lead); Writing‐review & editing (equal).

## Supporting information

Fig S1

Fig S2

Table S1

## Data Availability

The data that support the findings of this study are available from the corresponding author upon reasonable request.
