# Supplementary material for: Upregulation of the novel lncRNA U731166 is associated with migration, invasion and vemurafenib resistance in melanoma
Source: J Cell Mol Med. 2022 Jan 18;26(3):671–83. doi: 10.1111/jcmm.16987 (PMC8817119; doi:10.1111/jcmm.16987)
Supplement: Supplementary file 3 — Table S1 [file JCMM-26-671-s002.docx]

**Appendices**

| Supplementary Table 1. RNA binding proteins experimentally verified interacting with the lncRNA U73166.† | | | | | | | |  |
| --- | --- | --- | --- | --- | --- | --- | --- | --- |
| **RBP** | **clusterNum** | **clipExpNum** | **clipIDnum** | **HepG2** | **K562** | **pancancerNum** | **R** | **pvalue** |
| U2AF2 | 10 | 10 | 20 | NA | NA | 19 | 0.029 | 0.54 |
| **SRSF1** | **8** | **7** | **11** | **NA** | **NA** | **21** | **0.28** | **9.3e−10** |
| TAF15 | 7 | 7 | 12 | NA | NA | 22 | 0.094 | 0.044 |
| HNRNPA1 | 8 | 6 | 13 | NA | NA | 14 | 0.15 | 0.0015 |
| **HNRNPA2B1** | **12** | **6** | **22** | **NA** | **NA** | **22** | **0.3** | **9.2e−11** |
| CSTF2T | 11 | 4 | 22 | NA | NA | 17 | 0.13 | 0.0072 |
| DDX54 | 4 | 4 | 5 | NA | NA | 16 | −0.042 | 0.37 |
| DGCR8 | 5 | 4 | 5 | NA | NA | 26 | 0.2 | 1.1e−05 |
| HNRNPU | 9 | 4 | 15 | NA | NA | 19 | 0.18 | 8.2e−05 |
| **RBFOX2** | **5** | **4** | **8** | **NA** | **NA** | **16** | **0.27** | **3.3e−09** |
| HNRNPK | 2 | 3 | 3 | NA | NA | 16 | 0.16 | 0.00039 |
| HNRNPUL1 | 6 | 3 | 6 | NA | NA | 12 | 0.027 | 0.56 |
| PRPF8 | 3 | 3 | 4 | NA | NA | 12 | 0.039 | 0.41 |
| SLTM | 4 | 3 | 5 | NA | NA | 26 | 0.33 | 3.2e−13 |
| ADAR | 3 | 2 | 4 | NA | NA | 17 | 0.061 | 0.19 |
| AIFM1 | 3 | 2 | 3 | NA | NA | 12 | −0.079 | 0.09 |
| BUD13 | 6 | 2 | 6 | NA | NA | 19 | 0.083 | 0.074 |
| EIF4A3 | 8 | 2 | 9 | 2.027 | NA | 15 | −0.045 | 0.34 |
| ELAVL1 | 2 | 2 | 4 | NA | NA | 14 | 0.13 | 0.0071 |
| FAM120A | 7 | 2 | 8 | NA | NA | 13 | 0.11 | 0.019 |
| FBL | 4 | 2 | 4 | NA | NA | 16 | −0.094 | 0.043 |
| GTF2F1 | 2 | 2 | 3 | NA | NA | 11 | 0.11 | 0.016 |
| LIN28 | 3 | 2 | 3 | NA | NA | 20 | −0.045 | 0.34 |
| LSM11 | 2 | 2 | 2 | NA | NA | 21 | 0.065 | 0.16 |
| NONO | 3 | 2 | 3 | NA | NA | 16 | 0.12 | 0.011 |
| NUMA1 | 8 | 2 | 10 | NA | NA | 25 | 0.0019 | 0.97 |
| PCBP2 | 2 | 2 | 4 | NA | NA | 13 | 0.17 | 3e−04 |
| RANGAP1 | 3 | 2 | 4 | NA | NA | 19 | −0.049 | 0.3 |
| SAFB2 | 4 | 2 | 5 | NA | NA | 26 | 0.12 | 0.0081 |
| TIA1 | 1 | 2 | 2 | NA | NA | 26 | 0.24 | 2.4e−07 |
| U2AF1 | 2 | 2 | 2 | NA | NA | 23 | 0.22 | 1.2e−06 |
| ZNF184 | 6 | 2 | 7 | NA | NA | 21 | 0.27 | 3.3e−09 |
| AUH | 3 | 1 | 3 | NA | NA | 13 | 0.012 | 0.79 |
| DKC1 | 3 | 1 | 3 | NA | NA | 12 | 0.12 | 0.008 |
| FMR1 | 2 | 1 | 2 | NA | NA | 19 | 0.13 | 0.0048 |
| FUS | 1 | 1 | 1 | NA | NA | 25 | 0.13 | 0.0042 |
| IGF2BP2 | 1 | 1 | 1 | NA | NA | 20 | 0.27 | 2.5e−09 |
| ILF3 | 1 | 1 | 1 | NA | NA | 26 | 0.22 | 2.7e−06 |
| NOP56 | 3 | 1 | 3 | NA | NA | 15 | 0.14 | 0.0036 |
| NOP58 | 3 | 1 | 3 | NA | NA | 21 | 0.27 | 2.4e−09 |
| RBM10 | 1 | 1 | 1 | NA | NA | 22 | 0.1 | 0.028 |
| SMNDC1 | 1 | 1 | 1 | NA | NA | 14 | 0.13 | 0.0049 |
| SRSF10 | 1 | 1 | 1 | NA | NA | 25 | 0.25 | 4.3e−08 |
| SRSF9 | 1 | 1 | 1 | NA | NA | 16 | 0.23 | 3.4e−07 |
| VIM | 1 | 1 | 1 | NA | NA | 14 | 0.0093 | 0.84 |
| XRN2 | 2 | 1 | 2 | NA | NA | 12 | 0.26 | 1.7e−08 |
| †The gray highlight show shortlisted RBPs | | |  |  |  |  |  |  |
